# Effects of tumor treating fields (TTFields) on glioblastoma cells are augmented by mitotic checkpoint inhibition

**DOI:** 10.1038/s41420-018-0079-9

**Published:** 2018-07-16

**Authors:** Almuth F. Kessler, Greta E. Frömbling, Franziska Gross, Mirja Hahn, Wilfrid Dzokou, Ralf-Ingo Ernestus, Mario Löhr, Carsten Hagemann

**Affiliations:** Department of Neurosurgery, University of Würzburg, Tumorbiology Laboratory, Würzburg, Germany

## Abstract

Tumor treating fields (TTFields) are approved for glioblastoma (GBM) therapy. TTFields disrupt cell division by inhibiting spindle fiber formation. Spindle assembly checkpoint (SAC) inhibition combined with antimitotic drugs synergistically decreases glioma cell growth in cell culture and mice. We hypothesized that SAC inhibition will increase TTFields efficacy. Human GBM cells (U-87 MG, GaMG) were treated with TTFields (200 kHz, 1.7 V/cm) and/or the SAC inhibitor MPS1-IN-3 (IN-3, 4 µM). Cells were counted after 24, 48, and 72 h of treatment and at 24 and 72 h after end of treatment (EOT). Flow cytometry, immunofluorescence microscopy, Annexin-V staining and TUNEL assay were used to detect alterations in cell cycle and apoptosis after 72 h of treatment. The TTFields/IN-3 combination decreased cell proliferation after 72 h compared to either treatment alone (−78.6% vs. TTFields, *P* = 0.0337; −52.6% vs. IN-3, *P* = 0.0205), and reduced the number of viable cells (62% less than seeded). There was a significant cell cycle shift from G1 to G2/M phase (*P* < 0.0001). The apoptotic rate increased to 44% (TTFields 14%, *P* = 0.0002; IN-3 4%, *P* < 0.0001). Cell growth recovered 24 h after EOT with TTFields and IN-3 alone, but the combination led to further decrease by 92% at 72 h EOT if IN-3 treatment was continued (*P* = 0.0288). The combination of TTFields and SAC inhibition led to earlier and prolonged effects that significantly augmented the efficacy of TTFields and highlights a potential new targeted multimodal treatment for GBM.

## Introduction

Malignant gliomas are the most prevalent, highly aggressive, invasive, and difficult to treat primary brain tumors in adults. The standard treatment regimen for patients with glioblastoma multiforme (GBM), a World Health Organization (WHO) grade IV glioma^1^, includes microsurgical tumor resection followed by local radiation and chemotherapy with temozolomide^2,3^. However, in spite of this multimodal approach the prognosis is unfavorable with a median overall survival (OS) of around 16 months, a progression-free survival of 6.9 months and a 5-year survival of only 9.8%^4,5^. This is accompanied by severe deteriorations of the patients’ neurological and general conditions that impair their quality of life (QoL). Therefore, more efficient treatment options with lower side effects are urgently needed to improve the outcome of patients.

Tumor treating fields (TTFields) at 200 kHz are a novel approved GBM treatment modality that demonstrated an improved median OS by 4.9 months in newly diagnosed GBM patients with only minor side effects in a clinical phase III trial^6^ and no deterioration in QoL^7,8^. These alternating electric fields have a frequency range of 100–300 kHz and a field intensity of 1–3 V/cm. For clinical use they are applied at tumor specific frequencies via ceramic electrodes, so-called transducer arrays, adhered to the shaved scalp of the patient. The therapy compliance was tightly linked to the outcome and monthly compliance above 75% was associated with higher overall survival^9,10^. In addition, this important development in the treatment of GBM patients, the therapy may be further improved by its combination with synergistic therapies. To identify new facilitating compounds, we had a deeper look into the TTFields’ mode of action. TTFields interfere with mitotic processes of cells on subcellular level by inhibition of spindle fiber formation and influencing other dipole macromolecules essential for cell division such as septins, ultimately leading to mitotic catastrophe, which could culminate in cell death^11,12^. Further affected biological mechanisms involve apoptosis, autophagy, DNA repair, and immunogenic cell death^11–13^.

Recently, we showed that the inhibition of the spindle assembly checkpoint (SAC) by a crucial SAC regulator, i.e., the evolutionary conserved protein kinase monopolar spindle 1 (MPS-1, also known as TTK)^14^, efficiently reduces GBM cell proliferation in combination with a spindle toxin^15^. The SAC controls the fidelity of bipolar sister chromatid attachment to functional spindle microtubules, alignment of chromosomes at the metaphase plate and presence of spindle fiber tension to ensure equal sister chromatid segregation to daughter cells during mitosis^16^. Defects in these processes are detected by the SAC, which initiates a mitotic cell cycle arrest by blocking the progression of metaphase to anaphase^16^. A defective SAC results in chromosomal instability, aneuploidy and subsequent tumorigenesis^17^. However, in combination with spindle fiber damaging agents like the chemotherapeutic vincristine, it accelerates mitotic catastrophe, causes cell death and even leads to shrinkage of GBM tumors in a mouse model^15^. Therefore, it sensitizes GBM cells to the effects of antimitotic drugs and we hypothesized that the antimitotic effects of TTFields, partially mediated by disruption of the spindle apparatus, may be facilitated and enhanced by an inhibition of the SAC regulator MPS-1. Here, we investigated if the efficacy of TTFields would be augmented by a combination of TTFields that physically damage the spindle apparatus and chemical inhibition of the SAC, leading to earlier and prolonged effects.

## Results

### TTFields impair cell proliferation most efficiently at 200 kHz in various human glioblastoma cell lines

**Fig. 1:**
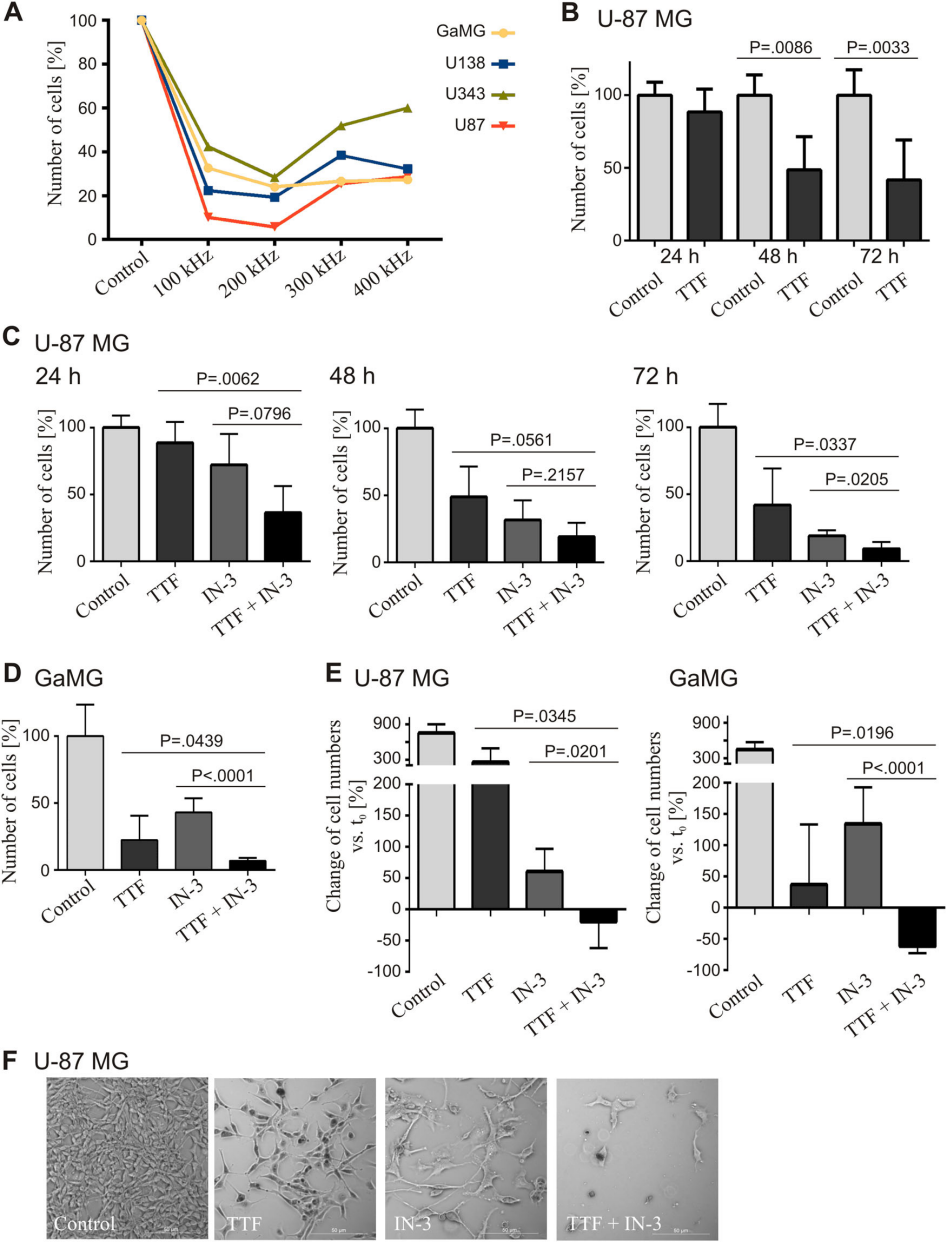
TTFields have antiproliferative effects, which are enhanced by mitotic checkpoint inhibition. Cells were treated with TTFields (TTF) and 4 µM of the MPS1 inhibitor MPS1-IN-3 (IN-3) either alone or in combination as indicated. **a** Determination of the optimal TTFields frequency for treatment of GBM cell lines in vitro. TTFields were applied for 72 h and the cells counted (*n* = 1). Totally, 200 kHz appeared to be the optimal frequency and was used for all further experiments. **b** Effect of TTFields (200 kHz) on U-87 MG cell numbers after 24, 48, and 72 h treatment. **c** U-87 MG cell numbers after 24, 48, and 72 h and (**d**) GaMG cell numbers after 72 h single and combined treatments as indicated. **e** Percentage change of U-87 MG (left) and GaMG (right) cell numbers after 72 h treatment compared to the 30,000 cells seeded at *t*_0_. **f** Phase contrast microscopy of U-87 MG cells after 72 h treatment (representative image of *n* = 3). If not otherwise stated, *n* ≥ 3 independently repeated experiments were performed. SD is shown as error bars

The TTFields frequency necessary to inhibit cell proliferation and to induce cell death is cell size-specific^11,18^. For GBM cells, a frequency of 200 kHz has been established and is applied in the clinical setting^11^. To reproduce these findings and to establish the in vitro technique in the laboratory, frequencies of 100, 200, 300, and 400 kHz were applied for 72 h to the four different human GBM cell lines GaMG, U-138 MG, U-343 MG, and U-87 MG grown as monolayers on coverslips. Compared to untreated control cells, all tested cell lines responded with a significant reduction in cell proliferation at all analyzed TTFields frequencies, as estimated by cell counts (Fig.1a). The maximum effect on cell proliferation was observed at 200 kHz, which is in line with previously published data^11,12,18^. Therefore, we proceeded at this specific frequency in all following experiments. U-87 MG cells were treated with TTFields for 24, 48, and 72 h. While there was no effect after 24 h, compared to the control, we observed significantly reduced cell numbers after 48 h (49%, *P* = 0.0086) and 72 h (42%, *P* = 0.0033), respectively, indicating a clear inhibitory effect of TTFields on proliferation (Fig. 1b, Supplementary Fig. 1A).

### The antiproliferative effect of TTFields is enhanced by SAC inhibition

Recently, we developed the SAC inhibitor MPS1-IN-3 (IN-3) and showed that it augments the efficacy of the microtubule destabilizer vincristine^15^. We hypothesized that we could achieve a comparable effect by combining IN-3 with TTFields, because one mechanism by which TTFields disrupts cell division is through the inhibition of spindle fiber formation. Therefore, U-87 MG cells were treated with IN-3, TTFields, or TTFields in combination with IN-3. Cells were counted after 24, 48, and 72 h **(**Fig. 1c, Supplementary Fig. 1C). There was no effect on cell numbers with TTFields and IN-3 alone after 24 h but an immediate and strong impact was with the TTFields/IN-3 combination. After 48 h of single treatment with IN-3 or TTFields, cell numbers were reduced to 49% (*P* = 0.0087, TTFields) and 32% (*P* = 0.0005, IN-3), respectively. The combination further decreased the cell numbers to 19% (*P* < 0.0001), each compared to the control. The cell number decrease culminated after 72 h at 9% in the combination compared to TTFields alone (42%, *P* = 0.0337) and to IN-3 alone (19%, *P* = 0.0205) (Fig. 1c, Supplementary Fig. 1C). Similar results were obtained for GaMG cells after 72 h treatment. Compared to TTFields alone, the combined treatment reduced GaMG cell numbers by 69% (*P* = 0.0439) and compared to IN-3 by 84% (*P* ≤ 0.0001) (Fig. 1d, Supplementary Fig. 1B). When compared to the 30,000 cells initially seeded, after 72 h U-87 MG control cells on average grew by 754%, while the single treatments grew 256% (TTFields) and 61% (IN-3), respectively (Fig. 1e, **left**). GaMG displayed similar proliferation with 446% (control), 37% (TTFields), and 135% (IN-3), respectively (Fig. 1e, **right**). Interestingly, in the combination treatment U-87 MG cell numbers were 19% (Fig. 1e, **left**) and GaMG cell numbers were 62% lower than the seeded cell numbers (Fig. 1e, **right**), indicating an even net tumor cell reduction by combining SAC inhibition with TTFields application. This was also confirmed by phase contrast microscopy of U-87 MG cells. In addition to changes in cell numbers, the treated cells appeared to be enlarged and showed an altered phenotype especially in the combination treatment (Fig. 1f).

### TTFields in combination with IN-3 causes accumulation of nuclear abnormalities, affects cell cycle and increases apoptosis

**Fig. 2:**
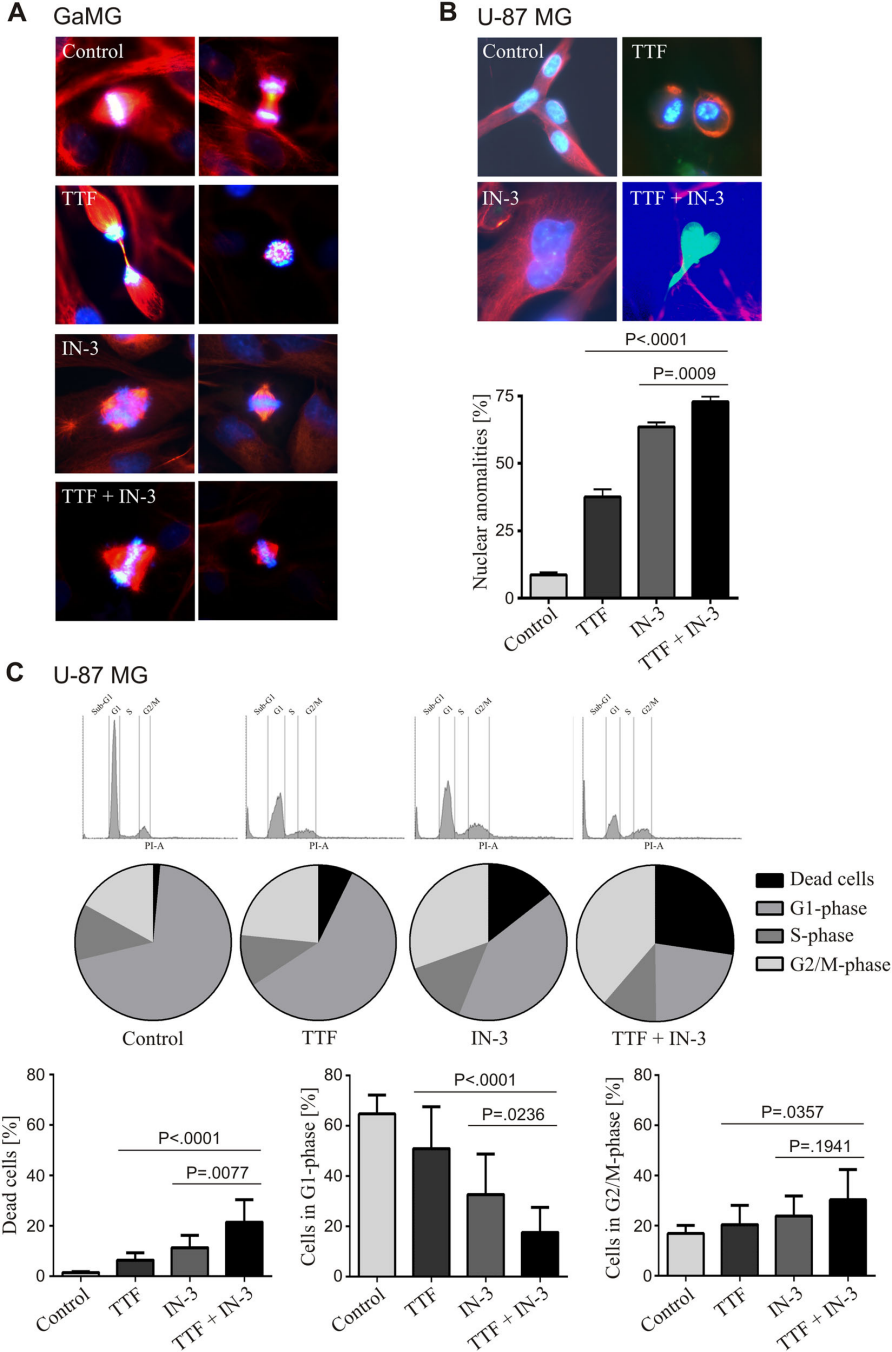
TTFields in combination with MPS1-inhibition affect the cell cycle and cause multipolar spindles and chromosomal missegregation. **a** Representative fluorescence images of typical mitotic figures of GaMG cells. The different treatments are indicated. Blue: DAPI, green: γ-tubulin, red: α-tubulin. **b** Representative fluorescence images of nuclear abnormalities (top) and their quantification (bottom) of U-87 MG cells. A total of *n* = 3 independent experiments were performed and of each experiment 100 nuclei were counted per treatment group. **c** Distribution of U-87 MG cells to the different cell cycle phases measured by FACS analysis (PI-staining). Histograms (top), average percentage distribution (middle) and percentage of cells in the sub-G1- (dead cells), G1- and G2/M-phase of the cell cycle are shown (*n* = 12). SD is shown as error bars

**Fig. 3:**
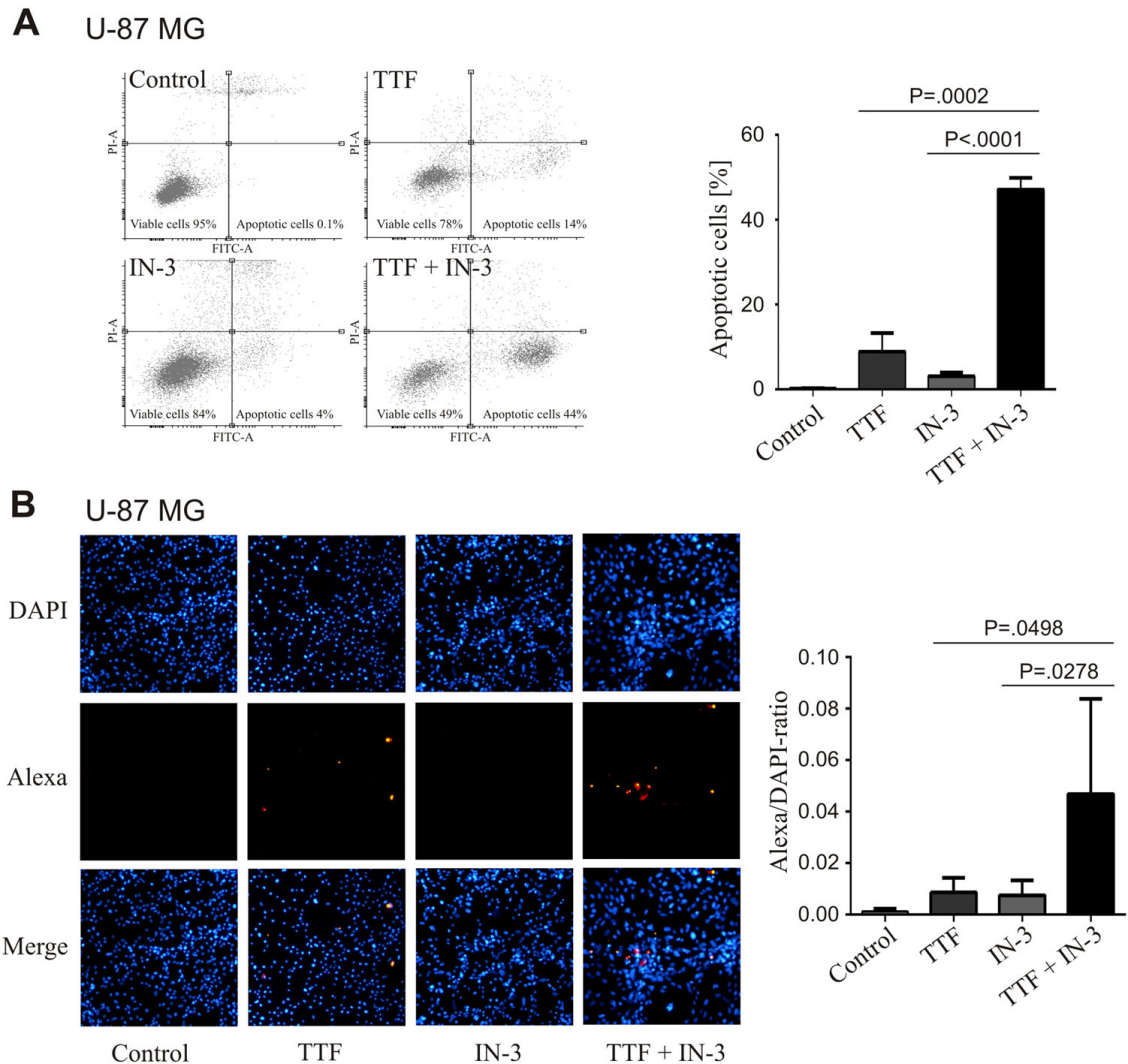
Increase of apoptotic cell death by the combined treatment with TTFields and IN-3 of U-87 MG cells. **a** FACS analysis of U-87 MG cells’ early apoptosis as measured by Annexin V staining after 72 h treatment as indicated. Representative histograms (left) and their quantification (right) are shown (*n* = 3). **b** Alexa-TUNEL assay of U-87 MG cells in situ to detect late apoptosis after 72 h treatment. Representative fluorescence images (left) and their quantification (right) are presented (*n* = 6). SD is shown as error bars

TTFields have been shown to disrupt mitosis and to increase abnormal mitotic figures^11,18^. Indeed, we observed very distinct mitotic figures, especially with the combined treatment, with multipolar spindles and massive chromosomal missegregation in GaMG cells (Fig. 2a). Subsequently, these disturbances lead to abnormal chromosome distribution, aneuploidy, and dysmorphic nuclei, as reported for both TTFields and IN-3^15,19^. Therefore, we quantified the number of abnormal nuclei in U-87 MG cells from the different treatment groups after 72 h **(**Fig. 2b). Both single treatments significantly increased the numbers of aberrant nuclei (TTFields: 38% and IN-3: 64%, both *P* < 0.0001) compared to the control (9%). The combined treatment led to the highest percentile of abnormal nuclei (73%), which was significantly higher than either treatment alone (*P* = 0.0002 vs. TTFields and *P* < 0.0001 vs. IN-3) (Fig. 2b). Further characterization of the cell cycle by FACS analyses clearly showed that the combination of TTFields with IN-3 caused a cell-cycle shift from mainly G1 to the G2/M-phase beyond the effects of the single treatment in U-87 MG (Fig. 2c) and GaMG cells (Supplementary Fig. 1D). In addition, a significant increase of sub-G1 cells was detectable, which most likely were dead cells subject to apoptosis (Fig. 2c, Supplementary Fig. 1D). The combination treatment of TTFields plus IN-3 induced an early stage of apoptosis in 44% of U-87 MG cells, compared to 14% with TTFields alone (*P* = 0.0002), and 4% with IN-3 alone (*P* < 0.0001) (Fig 3a). These data were confirmed by the TUNEL assay (Fig. 3b), and clearly showed that the inhibition of the SAC can considerably increase the effects of TTFields by enforcing cell death.

### SAC inhibition can bridge the interruption of TTFields treatment

**Fig. 4:**
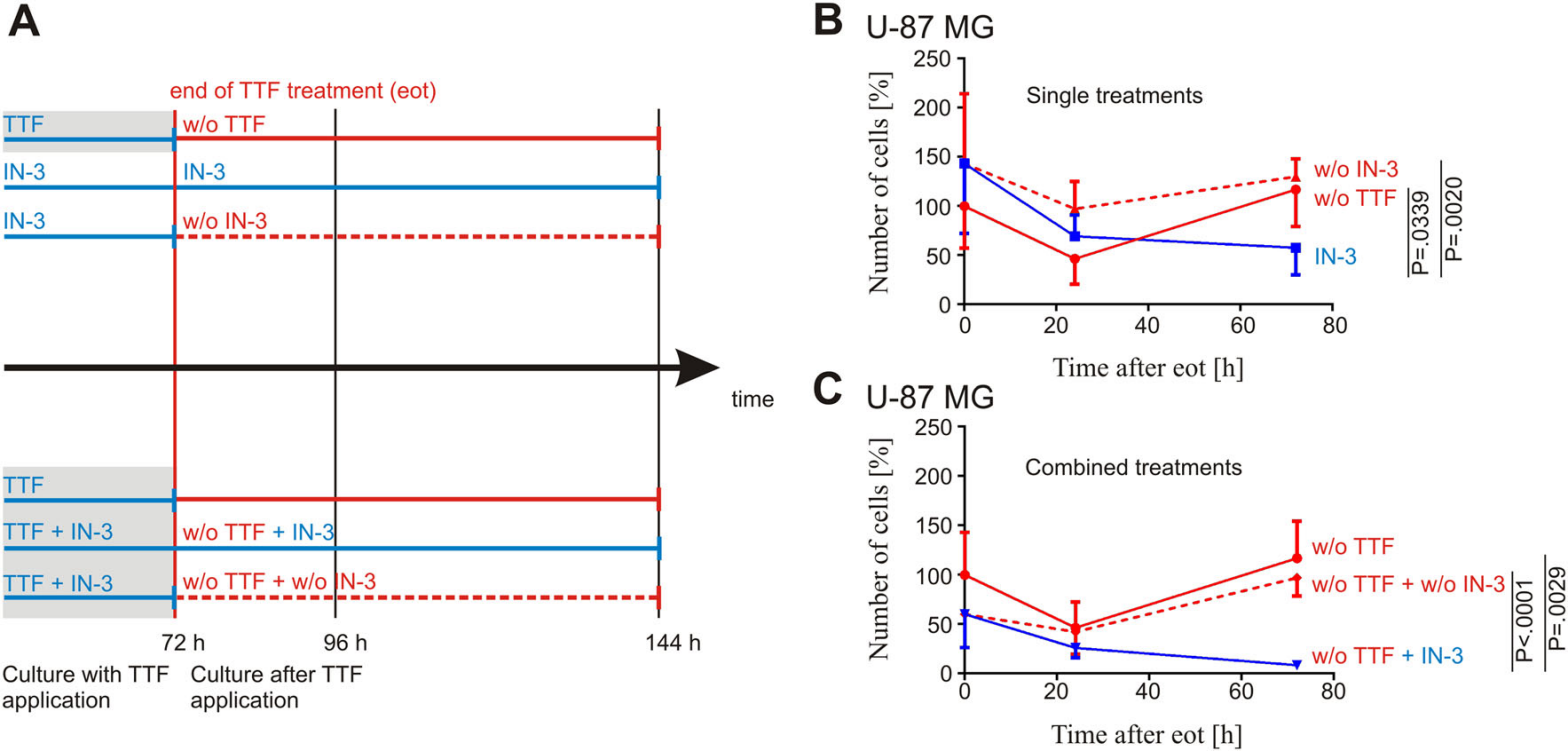
SAC inhibition prolongates TTFields-effects in U-87 MG cells. **a** Experimental scheme. Application of TTFields (gray background) and IN-3 is indicated in blue and was maintained either alone or in combination for 72 h. TTFields application was ended (EOT red) and cells cultured for another 72 h either with (IN-3, blue) or without (w/o IN-3, red, dotted line) IN-3. Cells were counted after 72, 96, and 144 h overall culture, as indicated. **b** TTFields were applied to U-87 MG cells for 72 h and then switched off. The cells’ proliferation was determined by cell counting at treatment end (0 h, 100%), and 24 and 72 h after end of TTFields application (w/o TTF, red). U-87 MG cells were incubated with 4 µM IN-3 for 72, 96, and 144 h (IN-3, blue) and counted or incubated with IN-3 for 72 h, further cultivated for 24 and 72 h without IN-3 (w/o IN-3, red, dotted line) and then counted. **c** For combined treatment, TTFields were applied to U-87 MG cells for 72 h and then switched off (w/o TTF, red), while 4 µM IN-3 was present for 72, 96, and 144 h, respectively (IN-3, blue) or cells were cultured for 72 h with TTFields and 4 µM IN-3 and then TTFields were switched off and IN-3 was removed, while the cells were further cultivated for 24 and 72 h after end of treatment (red, dotted line). Experiments were independently repeated with *n* ≥ 3. SD is shown as error bars

The combination of TTFields and IN-3 showed a more pronounced effect on cell proliferation (Fig. 1c), viability **(**Fig. 1e**)**, the ratio of dead/alive cells (Fig. 2c), and an increased apoptotic rate **(**Fig. 3**)** compared to the single treatments. An inevitable question is the sustainability of the applied treatments, especially whether the strong impact of the combination would translate into persistent and long-lasting effects. Therefore, we evaluated cell numbers after 72 h of exclusive TTFields treatment. Subsequently, the treatment was discontinued (end of treatment, EOT) and the cells proliferation observed for another 24 and 72 h **(**Fig. 4a**)**. Following EOT, cell numbers decreased significantly to 46% (*P* = 0.0400) during the next 24 h. However, 72 h after EOT the cells had recovered and restarted proliferation. For the treatment solely with IN-3 we found similar results **(**Fig. 4b**)**. Continued treatment with IN-3 for 72, 96, and 144 h led to a considerably reduced cell number to 59% (*P* = 0.0042), whereas a discontinuation of the IN-3 treatment after 72 h did not cause a further decrease of cells. Surprisingly, continuation of IN-3 treatment after 72 h of combined TTFields plus IN-3 treatment for another 24 and 72 h induced a further reduction of cell numbers down to 8% (*P* = 0.0288). Notably, this effect was stronger than the effect of IN-3 that was given permanently to the cells reflecting a synergism with the clonogenic effect of TTFields even after discontinuation of the latter treatment **(**Fig. 4c**)**.

## Discussion

GBM therapy urgently needs new treatment approaches that will improve overall survival, while preserving patients’ QoL. TTFields are a new therapy approved by the FDA for newly diagnosed and recurrent GBM and are considered a fourth treatment modality that improves overall survival with a minimal impact on patients QoL^6–8^. In the clinical setting, an optimal frequency of 200 kHz has been established for the treatment of GBM, which is in accordance with previously published^11,12,18^ as well as our own data derived from cell culture experiments that show that GaMG cells are more sensitive to TTFields than U87-MG cells. The primary mechanism of action for TTFields is the disruption of the normal spindle microtubule assembly by decreasing the ratio between polymerized and total tubulin^12^. Such spindle fiber damage usually activates the SAC and induces cell cycle arrest in the G2/M-phase until the spindle defect is resolved^16^. Prolonged induction of the SAC is the means by which established, chemical microtubule poisons in cancer therapies such as vinca-alkaloids or taxanes work. Prolonged metaphase arrest often causes apoptotic cell death^20–22^. However, one major problem of such therapeutic interventions is that SAC activation and metaphase arrest are not permanent. Cells can escape by a mechanism called mitotic slippage, a mitotic exit without cytokinesis, leading to tetraploid cells. The fate of these cells can be post-slippage cell death by mitotic catastrophe or during a G1-arrest by senescence. Some cells, however, resume proliferation and become aneuploid^23,24^.

Treating cancer cells with TTFields led to an increase of mitotic apoptosis, nuclear abnormalities like polynucleation, micronucleation, and autophagy^12,13^, which are all hallmarks of mitotic catastrophe^23^. These effects were confirmed in our experiments when applying TTFields to GBM cell lines. However, our main objective was to further improve the treatment efficacy of TTFields by facilitating and enhancing their spindle disrupting effect. This objective was based on our recent observation that inhibition of the SAC key regulator MPS1 by a newly developed inhibitor MPS1-IN-3 (IN-3) in conjunction with the application of the spindle poison vincristine resulted in significantly less cell cycle arrest, and drastic nuclear aberrations, including lobed nuclei, multinucleated cells and micronuclei, which reflect gross chromosome segregation defects. In addition, the combination of IN-3 and vincristine led to almost complete tumor shrinkage and prolonged survival in orthotopic GBM mouse models^15^. Therefore, we concluded that selective MPS1 inhibition sensitizes GBM cells to the effects of antimitotic drugs, an assumption supported by data reported by other groups^25–28^.

Indeed, our data provide evidence that a combination of MPS1 inhibition and TTFields treatment of GBM cells elicited more than just additive effects. The antiproliferative benefit of the combination treatment started after 24 h, while the single treatments only began to be effective after 48 h. This reflects an acceleration of the comparably slow effect of TTFields action, which is dependent on the direction of the cell axis and the cell division rate^11,12,18^. Importantly, the combination treatment was the only one causing a net reduction of cells below the seeded cell number, while all other treatments only throttled cell proliferation. Whereas the latter findings were in line with published data on TTFields^11,18^ and MPS1 inhibitors^29,30^, the former discovery is a novel finding indicating that the combination may increase cell death by mitotic catastrophe, an assumption confirmed by cell cycle analysis, immunofluorescence imaging and apoptosis assays. Thus, these findings may open new perspectives for the treatment of GBM patients by augmenting the TTFields efficacy.

The compliance with TTFields therapy in the clinical trials was tightly linked to the survival outcomes; monthly compliance above 75% was associated with higher overall survival^9,10,31^. Our cell culture experiments revealed that the surviving cells recover with a delay of 24 h after end of TTFields application. This observation suggests that interruption of TTFields treatment for 24 h may still be bridged by the repercussion of the therapy^11^, whereas a treatment break of more than 24 h would result in resumed tumor growth. Therefore, longer treatment breaks should be avoided to allow optimal clinical outcome. Nevertheless, there are circumstances that inevitably lead to discontinuation of the therapy, e.g., skin irritations^32,33^. It would be of clinical importance to determine if such treatment breaks could be bypassed. After treatment with TTFields and IN-3 for 72 h the cell numbers further decreased considerably at 72 h after terminating TTFields application, indicating a persisting effect when applying the MPS-1 inhibitor. Therefore, such a combination could potentially bridge short breaks and ease the everyday life of patients at same or even better efficacy.

Taken together, the combination of TTFields with the chemical inhibition of SAC was able to reduce GBM cell proliferation, increase apoptosis and could potentially serve as a bridge for TTFields therapy interruption in the clinical setting. Recently, several potent MPS1 inhibitors have been developed^26,34–36^ and two of them, BAY1161909 and BAY1217389, are currently in phase I clinical trials^27,37^. Our data provide a rationale for the future clinical evaluation of combined therapies utilizing TTFields and MPS1 inhibitors in patients with GBM.

## Materials and methods

### Cell lines, cell culture and TTFields application

The human GBM cell lines U-87 MG, U-138 MG, and U-343 MG were purchased from Cell Lines Service (CLS, Eppelheim, Germany). The cell line GaMG was obtained from the Leibniz Institute DSMZ-German Collection of Microorganisms and Cell Cultures (DSMZ, Braunschweig, Germany). Cells were grown as reported elsewhere^38^ in 75 cm^2^ flasks (Corning, New York, NY, USA) at 37 °C in an atmosphere of 5.0% CO_2_ and 100% humidity.

Novocure’s inovitro™ laboratory research system for the treatment of cancer cells was used to administer TTFields to GBM cells in vitro as described by Porat et al.^39^. In brief, 24 h before start of TTFields application, cells were trypsinized and plated by placing 350 µl medium containing 30,000 cells as a drop in the center of a glass coverslip (20 mm in diameter) (Hartenstein, Würzburg, Germany) within an inovitro ceramic dish (Novocure, Haifa, Israel). After 20 h incubation at 37 °C and 5.0% CO_2_ to allow the cells’ adhesion, the medium was removed and the plates were filled with 2 ml fresh medium and 2 ml medium containing 4 µM of MPS1-IN-3 (IN-3) (Sigma-Aldrich, St. Louis, MO, USA), respectively. The ceramic dishes were placed onto a base plate connected to a TTFields generator. Each ceramic dish contains two pairs of electrodes perpendicular to each other. A sinusoid function generator and an amplifier integrated into the inovitro system generate alternating electric fields^13^. The medium was renewed every 48 h.

### Cell counting

TTFields were applied for up to 72 h. To evaluate their effects, cells were trypsinized after 24, 48, and 72 h of TTFields application as well as 24 and 72 h after ending TTFields treatment (EOT) by removing the medium, washing with phosphate-buffered saline (PBS) (Biochrom, Berlin, Germany) and adding 0.5 ml Trypsin/EDTA solution (Gibco, Eggenstein, Germany)^39^. The reaction was stopped by adding 1 ml of medium to each plate and cells were counted utilizing the Scepter 2.1 cell counter (Merck, Darmstadt, Germany).

### Fluorescence immunocytochemistry

Cells grown on coverslips were washed with PBS and fixed in 4% (vol/vol) paraformaldehyde (Merck, Darmstadt, Germany) in PBS for 30 min at room temperature. Cells were rinsed three times with 70 µl TBST (50 mM Tris (Roth, Karlsruhe, Germany), 150 mM NaCl (Merck, Darmstadt, Germany), pH 8.0, 0.5% (vol/vol) Tween-20 (Sigma-Aldrich, St. Louis, MO, USA)) permeabilized and blocked in 70 µl blocking solution (10% (vol/vol) goat serum (Jackson, West Baltimore Pike, USA), 1% (wt/vol) BSA (Serva, Heidelberg, Germany), 0.05% (vol/vol) Triton-X 100 (Sigma-Aldrich, St. Louis, MO, USA) in PBS) for 30 min at room temperature. Totally,70 µl primary antibody mixture of rabbit anti γ-tubulin diluted 1:1000 and mouse anti α-tubulin diluted 1:2000 (both from Sigma-Aldrich, St. Louis, MO, USA) in 1% (wt/vol) BSA, 0.05% (vol/vol) Triton-X 100 in PBS, were added to each cover slip and incubated over night at 4 °C for immunocytochemistry. The cells were washed three times with 70 µl TBST and blocked for 30 min at room temperature in 70 µl blocking solution before they were incubated in the dark with the secondary antibody mixture Cy2-goat-anti rabbit diluted 1:50 and Cy3-goat-anti mouse (both from Jackson, West Baltimore Pike, USA) diluted 1:100 in 1% BSA, 0.05% Triton-X 100 in PBS. After 2 h incubation at room temperature cells were washed three times with 70 µl TBST. Cover slips were mounted to glass slides using fluoromount aqueous mounting medium containing DAPI (Sigma-Aldrich, St. Louis, MO, USA), dried over night at room temperature and stored for 24–48 h at 4 °C. Cells were viewed on an inverted fluorescence microscope LEICA DMI 3000 B. 100 nuclei of each treatment group were inspected and the ratio of aberrant to normal nuclei was calculated. Images were captured through a 100× objective by using the LEICA DFC450 camera and LAS V4.5 software (all Leica, Wetzlar, Germany).

### Cell cycle analysis and apoptosis assays

After 72 h of TTFields treatment, floating cells from the medium were harvested by centrifugation at 230×*g* and adherent cells were dissolved by trypsinization. Both cell populations were washed once with 5 ml ice cold PBS, combined and finally resuspended in 500 µl PBS.

For cell cycle analysis the cells were fixed in 4 ml ice cold 70% ethanol (J. T. Baker, Deventer, The Netherlands) and stained with propidium iodide (Sigma-Aldrich, St. Louis, MO, USA). Analysis of the DNA content was performed by flow cytometry (BD FACS Canto 2.0, Becton-Dickinson, Franklin Lakes, NJ, USA) and evaluated with Flowing Software 2.5.1 (University of Turku, Finland).

To measure cell death by Annexin V staining, PBS-washed cells were incubated for 15 min in 500 µl binding buffer (0.01 M HEPES pH 7.4 (Roth, Karlsruhe, Germany), 0.14 M NaCl, 2.5 mM CaCl_2_ (both from Merck, Darmstadt, Germany) in PBS), 10 µl propidium iodide (Sigma-Aldrich, St. Louis, MO, USA) and 5 µl FITC-Annexin (Beckton-Dickinson, Franklin Lakes, NJ, USA), at room temperature. Within 1 h measurements were performed by flow cytometry (BD FACS Canto 2.0, Becton-Dickinson, Franklin Lakes, NJ, USA). For cell death analysis by TUNEL assay, ethanol fixed cells were stained using the TUNEL Assay Kit—In situ Direct DNA Fragmentation (Abcam, Cambridge, UK) according to the manufacturers protocol. Photographs of stained cells were taken using the LEICA DFC450 camera mounted to a LEICA DMI 3000 B fluorescence microscope and LAS V4.5 software (all from Leica, Wetzlar, Germany). Apoptosis was quantified by counting DAPI and Alexa stained cells using ImageJ^40^ and calculating the DAPI/Alexa ratio.

### Statistical analysis

All experiments have been repeated independently at least three times, except for the test of the optimal TTFields frequency, which has been done only once as a proof of already published data^11,12,18^. Statistical analysis was performed using GraphPad Prism 6 Software (GraphPad Software Inc., San Diego, USA). Statistical significance was defined by unpaired two tailed Student’s *t* tests and ANOVA, as applicable. *P* < 0.05 was considered to be significant.

## Electronic supplementary material

Suppl. Figure 1

Supplementary figure legends

## Electronic supplementary material

**Supplementary Information** The online version of this article (10.1038/s41420-018-0079-9) contains supplementary material, which is available to authorized users.
