# Supplementary figures and images for: Effects of tumor treating fields (TTFields) on glioblastoma cells are augmented by mitotic checkpoint inhibition
Source: Cell Death Discov. 2018 Jul 16;4:77. doi: 10.1038/s41420-018-0079-9 (PMC6125382; doi:10.1038/s41420-018-0079-9)

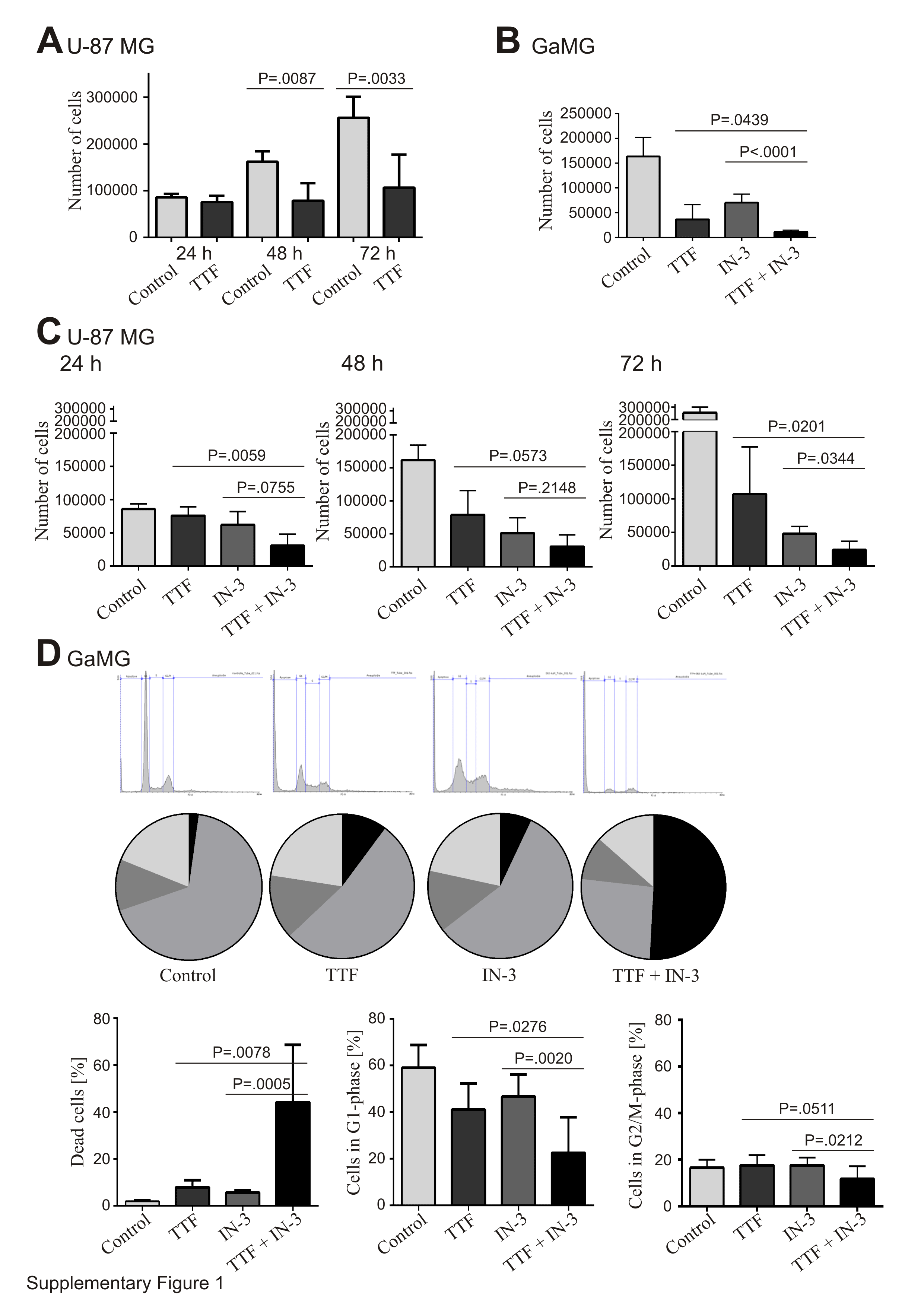

Supplement: Supplementary file 1 — Suppl. Figure 1 [file 41420_2018_79_MOESM1_ESM.tif]
