# Supplementary material for: Effects of tumor treating fields (TTFields) on glioblastoma cells are augmented by mitotic checkpoint inhibition
Source: Cell Death Discov. 2018 Jul 16;4:77. doi: 10.1038/s41420-018-0079-9 (PMC6125382; doi:10.1038/s41420-018-0079-9)
Supplement: Supplementary file 2 — Supplementary figure legends [file 41420_2018_79_MOESM2_ESM.docx]

## Supplementary Figure 1

Cells were treated with TTFields (TTF) and 4 µM of the MPS1 inhibitor MPS1-IN-3 (IN-3) either alone or in combination as indicated. A) Effect of TTFields (200 kHz) on absolute U-87 MG cell numbers after 24 h, 48 h and 72 h treatment. B) Absolute GaMG cell numbers after 72 h and C) absolute U-87 MG cell numbers after 24 h, 48 h and 72 h single and combined treatments as indicated. D) Distribution of GaMG cells to the different cell cycle phases measured by FACS analysis (PI-staining). Histograms (top), average percentage distribution (middle) and percentage of cells in the sub-G1- (dead cells), G1- and G2/M-phase of the cell cycle are shown. All experiments were independently repeated with n ≥ 3. SD is shown as error bars.
